# Termite baiting—how it changed the landscape of the pest management industry and termite research in Southeast Asia

**DOI:** 10.1093/jee/toaf081

**Published:** 2025-04-13

**Authors:** Chow-Yang Lee, Shao-Hung Dennis Lee

**Affiliations:** Department of Entomology, University of California, Riverside, CA, USA; Department of Entomology, University of California, Riverside, CA, USA

**Keywords:** Heterotermitidae, *Coptotermes gestroi*, survey, pest management industry, chitin synthesis inhibitor

## Abstract

The management of subterranean termite pests remains a major challenge in Southeast Asia, where these pests cause significant structural and economic damage. Termite baiting has emerged as an effective option to conventional soil termiticides, offering a safer pest management approach with reduced chemical input into the environment. In this paper, we review the history of termite research in Southeast Asia, highlighting the turning points of termite research, from agriculture and plantations to buildings and structures, and the transformative impact of termite baiting on the pest management industry in the region over the last 25 yr. We also discuss the outcome of a survey of pest management professionals on their baiting practices, bait performance, and reinfestation rates. All bait products eliminated termite colonies. There were significant differences in terms of the baiting period to colony elimination, with Xterm outperforming Sentricon, Exterra, and Exterminex. Above-ground (AG) baiting was preferred over in-ground (IG) baiting due to construction constraints and low IG station interception rates. While bait effectively controlled *Coptotermes* spp., challenges persist in managing fungus-growing termites such as *Macrotermes gilvus* Hagen. Reinfestation occurred in < 10% of baited premises.

## Introduction

Situated in the tropics, Southeast Asia, which consists of 11 countries (Brunei, Cambodia, Indonesia, Laos, Malaysia, Myanmar, Philippines, Singapore, Thailand, Timor-Leste, and Vietnam), has exceptional termite biodiversity. Published literature estimates the presence of approximately 180 species in Peninsular Malaysia (Tho 1992, Lee 2007a), 158 species in Thailand (Lertlumnaphakul et al. 2022), 200 species in Indonesia (Subekti and Milanio 2023), 141 species in Vietnam (Trinh et al. 2010), and 54 species in the Philippines (Acda 2004, 2013). While most of these species play ecological roles in nutrient cycling and ecosystem functioning, a fraction are recognized as major insect pests, causing damage to buildings, infrastructure, agriculture, and forestry. Among these, subterranean termites of the family Heterotermitidae (Hellemans et al. 2024), particularly those from the genus *Coptotermes*, represent the most important pests targeted by the pest management industry.

The economic burden of subterranean termite infestations in Southeast Asia is substantial. Termite management accounted for the largest share of revenue of pests targeted by the regional pest management industry (Lee 2014). Reports from Thailand (US$500 million annually; Vongkaluang 2004), Indonesia (US$535.6 million annually; Nandika 2015), and Malaysia (US$50 million annually; Lee and Yap 2003) highlight the financial impact of termite-related damages. Collectively, these costs are estimated to exceed US$1 billion annually for building owners across the region.

Termite baiting is a popular post-construction remedial treatment against subterranean termites in Southeast Asia (Lee 2014). Unlike soil treatment using liquid termiticide that utilizes large quantities of active ingredients (Lee and Neoh 2023), baits could effectively manage subterranean termite colonies with minimal amounts of toxicants (Rust and Su 2012, Evans and Iqbal 2015, Su 2019, Dhang 2024). The first termite bait system, Sentricon, containing 0.5% hexaflumuron, was introduced in Southeast Asian countries between 2000 and 2006. Soon thereafter, baiting became a popular option for termite management in Southeast Asia, and following Sentricon’s popularity were the introduction of several other baiting systems, such as Xterm (1% bistrifluron), Exterra (0.1% chlorfluazuron), Nemesis (0.1% chlorfluazuron), and Exterminex (0.1% chlorfluazuron). Anecdotally, it is said that today, for every 2 termite control jobs carried out in Southeast Asia, one is termite bait related.

This paper examines the impact of termite baiting on the pest management industry in Southeast Asia. We begin with an overview of termite research, tracing its historical development, the key factors that prompted investigations into termite infestations in buildings and structures, and the advancement of termite baiting in the region. We then review the outcome of a questionnaire survey conducted in 2023 among pest management companies based on baiting data collected between 2005 and 2023 to assess the baiting period and associated risks and discuss the implications of the survey results on termite baiting strategies and industry practices.

## Historical Account

Termite research in Southeast Asia began in the late 1800s to early 1900s, primarily focusing on taxonomy and its significance in forestry and agriculture for nearly a century (Harris 1957, Dhanarajan 1969, Tho 1992). The first recorded observations of termites in Malaysia were made by Haviland (1898), who described several new species. Bugnion (1913) published the first comprehensive list of Indo-Malayan termites, followed by Holmgren (1913–1914), who expanded the taxonomy based on Hugo Buttel-Reepen’s collections. John (1925) further enriched the literature with his observations of termite species across Sri Lanka, Peninsular Malaysia, Sumatra, and Java.

Rubber trees were introduced from Brazil to Malaya in 1877. Soon thereafter, *Termes gestroi* (now known as *Coptotermes gestroi* [Wasmann]) was found to be a major pest in the rubber plantations. This spurred significant research on pest control methods against plantation pests, as reflected in early works by Bailey (1901), Corey (1902), Ridley (1909), and Togwood (1909). Pratt (1908) provided a detailed ecological account of *T. gestroi* and other termite species affecting rubber plantations. The severity of infestations prompted a reward of £5000 (taking into account of inflation rate over the years, the value was estimated at US$174,544 in 2025) for effective control methods and the secondment of a scientific officer to solely study *T. gestroi* (Agricultural Bulletin of the Straits and Federated Malay States 1909). Later, control strategies included fumigation with sulfur arsenite mixtures (Richards 1915, 1917), followed by chlorinated hydrocarbons (Newsam and Rao 1958).

Termite problems gradually reduced in these plantations, and research on termites shifted to forest plantations, particularly conifer (*Pinus* spp.) and *Acacia mangium* Willd plantations (Tho 1974, Intachat and Kirton 1997, Kirton et al. 1999, 2000). Concurrently, interest in termite biology and ecology surged, resulting in numerous studies, especially after the mid-1970s (Gray and Dhanarajan 1974, Abe 1978, 1979, Abe and Matsumoto 1979).

Before 1949, there was no documented information on Thailand’s termite fauna. Snyder (1949) listed 6 species in his global catalog. Ahmad (1965) conducted the first comprehensive study, collecting nearly 400 colonies and identifying 74 species, 32 of which were new. Morimoto (1973) expanded the list to 90 species. Sornnuwat et al. (2004) analyzed over 4,300 samples collected between 1992 and 2004, identifying 178 species across 37 genera. Recently, Lertlumnaphakul et al. (2022) revised the total number of species to 158 based on extensive literature and database reviews.

In the Philippines, foundational work on termite taxonomy was carried out by Light (1921a, 1921b, 1929, 1930) and Oshima (1917, 1920). Light (1929) notably described *Coptotermes vastator* Light but it was later synonymized as *C. gestroi* (Yeap et al. 2007). Using 3 mitochondrial genes (COII, 12S, and 16S) from samples collected in Malaysia, Singapore, Thailand, Indonesia, Philippines, and Hawaii and morphometric measurements, Yeap et al. (2007) investigated the phylogenetic relationship between *C. gestroi* and *C. vastator.* They found that *C. vastator* and *C. gestroi* are synonymous, based on high sequence homology across the 3 genes. They concluded that *C. vastator* is a junior synonym of *C. gestroi*.

Similarly, Kirton and Brown (2003) also pointed out that *Coptotermes havilandi* Holmgren is a junior synonym of *C. gestroi*, while *C. borneensis* Oshima is a junior synonym of *Coptotermes travians* (Haviland). In addition, misidentifications were not uncommon and had occurred in many papers involving *Coptotermes* spp in Southeast Asia. Kirton and Brown (2003) reported that *C. travians* and *C. havilandi* had been misidentified in Peninsular Malaysia as *C. havilandi* and *C. travians*, respectively, due to description errors in Tho (1992). Because of the error, some earlier publications prior to 2003 that described *C. travians* were referring to *C. havilandi* (= *C. gestroi*) (eg Lee 2002a), while those of *C. havilandi* (= *C. gestroi*) were referring to *C. travians*.

## Turning Point of Termite Research and Pest Management Industry

Many housing developments in Southeast Asia are constructed on land previously utilized for plantations, where termites, particularly species of the genus *Coptotermes,* have long been significant pests in rubber, oil palm, and forest plantations. Tree trunks are removed during the land-clearing process for housing developments, but underground roots are often left behind. These residual roots serve as a food source for subterranean termites during construction. Once these natural food sources are depleted, termites migrate into buildings for resources. This issue is exacerbated by the common practice of burying wood debris from land clearing on-site rather than properly removing it, creating long-lasting termite food sources and heightening the risk of infestations in newly constructed structures (Kirton et al. 2000).

Before the 1990s, research on termites in Southeast Asia focused primarily on their impact on plantations and forestry, with limited information on termite infestations in buildings and urban structures. Two factors changed the focus of termite research to urban settings after the 1990s. The first factor was the ban on chlordane, a chlorinated hydrocarbon widely used as a soil termiticide from the 1950s to the 1990s. Chlordane was the preferred soil treatment for pre- and post-construction termite control due to its effectiveness and long residual activity (Lee and Chung 2003, Lee and Neoh 2023). However, its persistence and associated adverse effects on the environment have led to its eventual global restriction, beginning in the 1980s (EPA 1987, Lee and Neoh, 2023). In Southeast Asia, Brunei banned chlordane in 1980 (UNEP Chemicals 2002), Malaysia in 1998 (UNEP Chemicals 2002, IPEN 2005), Singapore in 1999 (UNEP Chemicals 2002), Philippines in 1999 (FPA 2025), Thailand in 2000 (PCD 2020), and Indonesia in 2001 (Mova Al’Afghani and Paramita 2018).

With the ban of chlordane use, pest management professionals (PMPs) in the region were left with alternative termiticides, such as chlorpyrifos, fenvalerate, cypermethrin, and lambda-cyhalothrin. These compounds, unlike chlordane, required precise application, and control failures became increasingly common (Lee and Neoh 2023). Many sought to learn more about termite biology and termite control strategies. However, existing literature from the United States and Europe at that time offered limited relevance due to differences in termite species and ecological contexts (Lee 2025). This highlighted a knowledge gap in understanding termite infestations in Southeast Asia.

The second factor was the introduction of termite baiting systems. Between 2000 and 2006, the Sentricon termite baiting system was introduced to Southeast Asia (Malaysia, Singapore, and Philippines in 2000, Thailand and Indonesia in 2003, and Brunei in 2006), followed by other bait systems. Baiting provided an effective and reliable option to the PMPs in Southeast Asia, who had long relied on liquid termiticide and dusting to treat termites at that time. However, one major challenge arose after the termite bait was introduced. Unlike in the United States and Europe, the region has high termite diversity. It is not uncommon to find buildings infested by multiple termite species simultaneously. While baiting systems demonstrated high efficacy against *C. gestroi* and *Coptotermes curvignathus* Holmgren, their effectiveness against non-heterotermitid species, such as fungus-growing termite *Macrotermes gilvus* Hagen, rhinotermitid *Schedorhinotermes medioobscurus* (Holmgren), and termitid *Globitermes sulphureus* (Haviland) and *Microcerotermes crassus* Snyder were unknown at that time. This gap created a demand for research on termite biology, ecology, and control strategies, prompting funding from industry and the government.

The introduction of termite baiting systems also transformed Southeast Asia’s pest management industry by allowing PMPs to charge higher for their services, resulting in higher profits and rapid industry expansion. In fact, some pest management companies abandoned soil treatment and only provide termite baiting in their termite control portfolio. Homeowners prefer termite baiting because, unlike soil liquid termiticide treatment that requires drilling of the floor of their homes, termite baiting is less intrusive and offers the potential to eliminate termite colonies. Besides, colony elimination with soil termiticides is not widely demonstrated (Lee and Neoh 2023). With exception to several studies (Potter and Hillery 2002, Vargo and Parman 2012, Keefer et al. 2012, Itakura et al. 2021), most published lab and field studies have shown that liquid termiticides such as fipronil were not able to eliminate termite colonies (Su 2005, Chouvenc 2018, 2024, Ripa et al. 2007, Henderson et al. 2016, Baker and Miguelena 2020).

It was also during this period that many Southeast Asian countries began requiring PMPs to obtain licenses to apply pesticides through environmental protection agencies, food and drug authorities, or equivalent regulatory bodies (Lee 2025). Licensing requirements included minimum educational qualifications; for example, in Malaysia, it requires completing secondary school education and passing the pesticide application examination administered by the Pesticide Control Division of the Department of Agriculture, which gradually phased out individuals with lower qualifications and limited training.

As pest management companies experienced higher profit margins, they could afford to recruit university graduates trained in entomology, integrated pest management, and pesticide science. This demand for entomology graduates grew in the early 2000s, catalyzed by licensing requirements for pesticide applicators. These trained professionals brought critical expertise to the industry as field biologists and marketing specialists (Lee 2025). Consequently, the pest management industry in Southeast Asia evolved into a more knowledge-driven sector, significantly advancing urban pest management practices.

## Termite Baiting and Related Research in Southeast Asia

Because of the high diversity of termites, multiple termite species are frequently encountered in and around buildings and structures (Table 1). Some species, especially those in the genus *Coptotermes*, are common pests infesting buildings, while others are detected at a lesser frequency (Sajap and Wahab 1997, Lee 2002b, Lee et al. 2007). Lee (2002b) reported that all infestations in the buildings surveyed (*n* = 32) in 1998 were caused by *Coptotermes* spp. Based on 167 termite samples collected in and around buildings, Kirton and Azmi (2005) reported the relative incidence of subterranean termite species in Peninsular Malaysia and Singapore. They found that *Coptotermes* spp. accounted for all the infestations in urban areas, while 64% were in semi-urban areas. The Asian subterranean termite, *C. gestroi,* is the most destructive species in Southeast Asia (Neoh and Lee 2023). It accounted for 85% of all building infestations in urban areas and 40% in suburban areas. Two other species of *Coptotermes*, namely *C. kalshoveni* and *C. curvignathus,* were found to have a lower frequency. The authors also revealed that there has been a decreased proportion of infestations of the latter 2 species between pre-1993 and 2004, while there was an increase in *C. gestroi* infestation. They attributed the decrease could be due to the inability of the latter 2 species to compete with *C. gestroi,* especially in the urban habitat, particularly after the conversion of agricultural land to cities and urban residential areas, reinforcing the notion that *C. gestroi* is an urban exploiter (Zhang and Evans 2017). Peridomestic species, especially termitids such as *M. gilvus*, *Mi. crassus*, *G. sulphureus*, *S. medioobscurus*, etc., accounted for ~15% of the infestations in buildings (Lee et al. 2007).

**Table 1.** Common subterranean termite pests in buildings and structures of Malaysia, Singapore, Thailand, and Indonesia (based on Sornnuwat et al. 1996, Sajap and Wahab 1997, Lee 2002b, Yunus et al. 2005) and their responses to termite baits.

Species	Characteristics			Responses to bait
	Structural pests	Perimeter species	Mound builder	
*Coptotermes gestroi*	Yes	Yes	No	Good
*Coptotermes curvignathus*	Yes	Yes	No	Good
*Coptotermes kalshoveni* Kemner	Yes	Yes	No	Good
*Coptotermes premrasmii* Ahmad	Yes	Yes	No	Good
*Macrotermes gilvus*	Sometimes	Yes	Yes (ground)	Poor
*Macrotermes carbonarius* (Hagen)	Rarely	Yes	Yes (ground)	Poor
*Microcerotermes crassus*	Sometimes	Yes	Yes (aboreal)	Moderate
*Nasutitermes havilandi* (Desneux)	Sometimes	Yes	Yes (aboreal)	Moderate
*Globitermes sulphureus*	Sometimes	Yes	Yes (ground)	Moderate
*Ancistrotermes pakistanicus* (Snyder)	No	Yes	No	Poor
*Schedorhinotermes medioobscurus*	Yes	Yes	No	Moderate
*Odontotermes* spp.	Rarely	Yes	No	Poor

Published studies on field evaluations of baits against different species of subterranean termites in Southeast Asia are shown in Table 2. In all studies (except for Neoh et al. 2011 and Lee et al. 2014, who investigated the fate of the termites in their mounds after baiting), colony elimination was inferred based on the absence of termites after they fed on the bait for several weeks. So far, baits containing 4 chitin synthesis inhibitors (0.5% hexaflumuron, 0.1% chlorfluazuron, 0.1 and 0.5% noviflumuron, and 0.5 and 1% bistrifluron) have been evaluated against several important species such as *C. gestroi*, *C. curvignathus*, *G. sulphureus,* and *M. gilvus.* The baits evaluated were in-ground bait (IG), above-ground bait (AG), or a combination of both. When tested against heterotermitids such as *C. gestroi*, colony elimination took ~4 to 13 wk, requiring as low as 0.02 g of toxicant. Lee (2007b) reported the elimination of *C. gestroi* colonies using 1% bistrifluron AG bait in 4 to 5 wk. On the other hand, when using 0.1% chlorfluazuron, 6 to 8 wk were taken to eliminate colonies of *C. curvignathus* (Peters et al. 2008, Sukartana et al. 2009). A more extended period was required to eliminate termitids although limited studies have been published in Southeast Asia, especially on fungus growing termites such as *M. gilvus*. Based on mere observation and the absence of empirical data, Peters and Broadbent (2005) and Peters et al. (2008) reported that 12 to 18 wk were required to eliminate colonies of *M. gilvus*, *Nasutitermes havilandi* (Desneux), *Nasutitermes luzonicus* Oshima and *Microcerotermes losbanosensis* Oshima using 0.1% chlorfluazuron bait.

**Table 2.** Field evaluation of termite baits against different species of subterranean termites in Southeast Asia.

Location	Species	% toxicant	Amount of toxicant used (g)	IG or AG^1^	Period taken to colony elimination	Reference
Malaysia	*Coptotermes curvignathus*	0.5% hexaflumuron	0.138–0.395	IG	25–44 d	Sajap et al. (2000)
		0.1% chlorfluazuron	0.47	IG	8 w	Peters et al. (2008)
	*Coptotermes gestroi*	0.5% hexaflumuron	0.92–1.46	IG	34–44 d	Lee (2002a)
		0.5% hexaflumuron	0.15–1.09	AG	35–62 d	Sajap et al. (2002)
		0.5% hexaflumuron	0.11–0.84	IG, AG	42–77 d	Sajap et al. (2009)
		0.1% noviflumuron	0.02–0.14	AG	46–78 d	Lee (2007a)
		0.5% noviflumuron	0.04–0.70	AG	41–80 d	Lee (2007a)
		0.5% noviflumuron	0.10–0.80	AG	35–46 d	Sajap et al. (2005)
		0.5% bistrifluron	1.06–3.23	AG	6–8 w	Lee (2007b)
		1.0% bistrifluron	1.73–8.34	AG	4–5 w	Lee (2007b)
		0.1% chlorfluazuron	0.22–0.30	IG	4–8.6 w	Umar and Majid (2020)
	*Ancistrotermes pakistanicus*	0.5% hexaflumuron	0.06	IG	54 d	Sajap et al. (2009)
	*Globitermes sulphureus*	1% bistrifluron	0.14–1.22	IG	8–16 w	Neoh et al. (2011)
	*Globitermes sulphureus*	0.1% chlorfluazuron	0.63	IG	11 w	Peters et al. (2008)
	*Macrotermes gilvus*	0.1% chlorfluazuron	0.54–0.68	IG	16 w^2^	Lee et al. (2014)
	*Nasutitermes havilandi*	0.1% chlorfluazuron	0.50–0.60	IG	12–18 w	Peters et al. (2008)
	*Schedorhinotermes* sp.	0.5% hexaflumuron	0.24	IG	59 d	Sajap et al. (2009)
Indonesia	*Coptotermes curvignathus*	0.1% chlorfluazuron	0.42–2.54	IG, AG	6–8 w	Sukartana et al. (2009)
Indonesia	*Coptotermes gestroi*	1.0% bistrifluron	1.2	AG	6 w	Yusuf et al. (2009)
Philippines	*Coptotermes gestroi*	0.1% chlorfluazuron	0.50–2.37	IG, AG	6–13 w	Garcia et al. (2007)
		0.1% chlorfluazuron	0.36–0.56	AG	8–12 w	Rojo and Acda (2019)
		0.1% chlorfluazuron	0.4	IG	8 w	Peters et al. (2008)
	*Macrotermes gilvus*	0.1% chlorfluazuron	2.6–3.2	IG	12–16 w	Peters and Broadbent (2005), Dhang (2011)
		0.1% chlorfluazuron	0.9–7.0	IG	6–18 w	Peters et al. (2008)
	*Microcerotermes losbanosensis*	0.1% chlorfluazuron	1.0	IG	17 w	Peters et al. (2008)
	*Nasutitermes luzonicus*	0.1% chlorfluazuron	0.75	IG	12	Peters et al. (2008)
Thailand	*Globitermes sulphureus*	0.1% chlorfluazuron	5.8–10.0	IG	16 w	Peters and Broadbent (2005)

^1^IG = in-ground baiting, AG = above-ground baiting.

^2^Inspection on medium size mounds showed reduction of 90–95% population, while inspections on large size mounds showed only 15–40% reduction. No colonies were eliminated.

On the contrary, Lee et al. (2014) reported variable performance of the same termite bait against medium and large field colonies of the fungus-growing termite *M. gilvus* in Malaysia. Their results showed that after 16 wk of baiting, bait performance differences were observed based on colony size: medium-sized colonies experienced a 90 to 96% population decline, queens became unhealthy, and larvae were absent, rendering colonies moribund. In contrast, large colonies saw only 15 to 40% population reduction, with healthy queens and larvae present. Gas chromatography-mass spectrometry (GC–MS) analysis revealed higher toxicant concentrations in medium colonies’ workers and fungus combs compared to large colonies. While chlorfluazuron effectively suppresses medium colonies, larger colonies may require prolonged baiting or additional bait stations. The unique biology of *Macrotermes*, including non-molting workers and reliance on fungus combs, limits the efficacy of chitin synthesis inhibitors. The authors attribute this disparity to the larger volume of fungus combs in big colonies, which dilutes chlorfluazuron concentration per unit comb, and the potential degradation of chlorfluazuron by symbiotic *Termitomyces* fungi, reducing toxicant transfer to larvae.

Chiu and Li (2024) reviewed the challenges of controlling fungus-growing termites (Macrotermitinae) using baiting systems. They identified 5 key biological and ecological barriers: (i) sporadic foraging behavior, leading to inconsistent bait consumption; (ii) naturally small colony sizes and high colony density, complicating targeted elimination; (iii) non-molting worker castes, rendering chitin synthesis inhibitors ineffective on workers and reliant on larval exposure; (iv) fungal symbiosis, where termite-cultivated *Termitomyces* fungi degrade toxicant or delay toxicant transfer to larvae; and (v) broad dietary preferences, reducing reliance on cellulose-based baits.

As there is a broader range of pest termite genera consisting of both heterotermitids and termitids in Southeast Asia, such a situation may pose a challenge to PMPs. The problem could be further complicated when a structure is attacked simultaneously by more than one species or in succession by a different species (Lee 2007a, Lee et al. 2007). In addition, when baiting suppresses or eliminates populations of heterotermitids such as *Coptotermes*, other species that are typically considered secondary pest species, such as *M. gilvus* and *G. sulphureus,* could enter and infest the structures afterward (Lee 2002a; Lee 2002b; Kirton and Azmi 2005; Lee et al. 2007). Although most reinfestations were caused by *Coptotermes* (83%) (Lee et al. 2007), PMPs find it challenging to explain to homeowners when the building is infested by non-*Coptotermes* spp that the baits were only effective against *Coptotermes* spp. The inconsistent performance of termite baits against fungus-growing termites often led to pest management professionals resorting to using liquid termiticides and physical removal of termite mounds when non-*Coptotermes* spp. were encountered (Lee et al. 2002a, 2002b, Lee et al. 2007a, b, 2014, Ngee et al. 2004).

Identifying an effective bait formulation and baiting procedure for managing fungus-growing termites remains one of Southeast Asia’s most pressing challenges in termite management. Future research should prioritize the optimization of bait matrices and toxicants to enable their effectiveness across different pest termite species and to reduce the baiting period (time to colony elimination), especially for fungus-growing termites such as *M. gilvus*. Achieving this requires a deep understanding of the nutritional ecology of these termitids. Another significant challenge in baiting these termitids is their sporadic foraging behavior, as described by Chiu and Li (2024). The authors proposed area-wide bait installation as a potential solution, which may be practical in plantation settings. However, its applicability to urban and structural pest management may be limited as it would result in higher treatment costs, prompting building owners to opt for liquid termiticide treatments instead.

## Termite Baiting Practices of Southeast Asian PMPs

A survey of termite baiting among pest management professionals in Southeast Asia on baiting strategies and industry practices (see Supplementary File) revealed that more than 80% of termite baiting interventions reported by PMPs utilized AG baiting systems. The preference for AG over IG baiting in Southeast Asia can be attributed to several factors. First, most people in the region live in high-rise apartments, condominiums, and terraced (linked) housing, which differs markedly from the single-family dwellings typical in other regions such as the United States. These urban structures pose significant challenges for installing IG bait stations, which require accessible soil. In contrast, AG baiting allows direct deployment at active infestation locations while enabling building owners to monitor bait stations throughout the treatment cycle, including during routine PMP inspections.

Second, the high biodiversity of termites along building perimeters complicates IG baiting. Beyond the targeted *Coptotermes* spp., numerous other termite species frequently colonize IG stations, reducing the chances of the primary pest species being intercepted. Furthermore, interference by diverse tropical soil-dwelling arthropods, particularly ants (Yeoh and Lee 2007), also exacerbates these challenges. Ants are known to disrupt termite foraging activity within bait stations, often displacing termite populations (Gulmahamad 1998; Scharf et al. 2002).

Third, the interception efficacy of IG stations for *C. gestroi* in Southeast Asia is notably low (2.1%), contrasting with the ~17% interception rate reported for *C. formosanus* in Florida (Su 1994). A recent study also corroborates this discrepancy. Su et al. (2023) observed that none of the 83 IG stations in Florida were intercepted by *C. gestroi*, necessitating AG bait deployment on infested trees for colony elimination. The persistently low interception rates of IG systems in Southeast Asia risk delaying the baiting process, which could result in building owners rescinding the contract; hence, PMPs are more incentivized to prioritize AG baiting.

One notable development is the introduction of Sentricon Recruit HD in-ground bait in several Southeast Asian countries—Indonesia and Malaysia prior to 2018 and Singapore in 2022 (Chee-How Tay, personal communication). Unlike conventional IG bait, whose cellulosic bait matrix degrades quickly in the ground, HD baits are highly durable and can withstand all the biotic and abiotic elements for more than 12 mo (Su 2007). Because of its durability, the bait could be installed in the ground for an extended period without first intercepting the termites followed by bait placement, unlike the conventional baiting procedure. This also minimizes the need for frequent visits for inspections of the monitoring bait stations, hence reducing the technician’s time and labor to visit the property for inspection. Besides, it also overcomes the issue of termites abandoning the station due to disturbance during the inspection process (Lee et al. 2007). However, despite these advantages, the use of HD bait in the region is limited due to several reasons: (i) Unlike in other parts of the world, satisfaction with a pest control service for a home or building owner in Southeast Asia depends on how quickly the problem is resolved and how frequent the pest management professional (PMP) return for follow-up visits. (ii) As mentioned, IG baits have a poor interception rate by the predominant species, *C. gestroi*. These 2 reasons explain why the use of HD bait remains low in the region. For HD bait to be successfully adopted for termite control in Southeast Asia, several things must happen. First, any existing termite infestation in the house should be eliminated using AG bait. Once the infestation is taken care of, HD bait can then be installed as a long-term termite management tool. Additionally, a change in consumer behavior is necessary, ensuring that homeowners or building owners do not expect frequent visits from PMPs.

The survey results demonstrated marked differences in speed of elimination among termite bait systems, with the Xterm system exhibiting the shortest mean baiting period (~3-fold elimination speed) compared to the Sentricon system. These disparities are likely due to variations in active ingredient and bait formulation. Xterm uses 1% bistrifluron, whereas Sentricon relies on 0.5% hexaflumuron. Notably, while the Sentricon system in the US has transitioned to the more active noviflumuron, the Sentricon system in Southeast Asia continues to use the older hexaflumuron-based formulation. Although field trials in Malaysia found no major difference in performance between hexaflumuron and noviflumuron baits against *C. gestroi* (see Table 2), laboratory data on other species, such as *Reticulitermes flavipes* showed the latter toxicant to be more active. Radiolabeled feeding assays revealed noviflumuron’s superior speed of action, potency, and retention (4-fold slower clearance) relative to hexaflumuron (Sheets et al. 2000, Karr et al. 2004). Field corroboration in the United States showed noviflumuron eliminated 74 *Reticulitermes* colonies in half the time (107 d vs. 205 d) required for hexaflumuron (Smith et al. 2001).

Xterm’s enhanced performance may be derived from 2 factors. First, bistrifluron shares noviflumuron’s higher bioactivity than hexaflumuron and chlorfluazuron, accelerating colony suppression. Second, its solid alpha-cellulose pellet size is larger than that of Sentricon, providing greater surface area, facilitating rapid exploration and consumption by termites (Evans and Gleeson 2006, Evans 2010). Bait matrix composition is known to play a major role in modulating efficacy. Early Sentricon systems in Southeast Asia used Laminated Textured Cellulose (LTC) matrix, which was effective only against *Coptotermes* spp. (Lee 2002a), with non-target species like *S. medioobscurus* and *A. pakistanicus* showing minimal attraction. Subsequent adoption of the present Preferred Textured Cellulose (PTC) matrix improved cross-species acceptance (Sajap et al. 2009). However, *C. gestroi* exhibited no preference between LTC and PTC, underscoring species-specific foraging behaviors.

Baiting programs in public buildings exhibited a prolonged colony elimination period compared to residential structures such as houses and apartments. This difference arises from 3 inter-related factors. First, the structural complexity of public buildings make termite inspections and bait placement challenging, as these facilities often contain concealed voids, utility closets, service ducts, and multi-level construction that hinder comprehensive access. Second, logistical challenges, such as coordinating with diverse stakeholders (eg security guards, facility managers), frequently delay bait installation and maintenance. Third, the larger footprint of these buildings increases the likelihood of concurrent infestations by multiple termite colonies, hence requiring wider coverage and more bait stations to target all active colonies. In contrast, houses and apartments typically present smaller, more contained environments with only 1 or 2 colonies, enabling more focused and expedited baiting interventions.

The survey data revealed that 9% of baited premises (1,794 out of 19,553 sites) experienced reinfestation after initial colony elimination. Premises treated with Sentricon were reinfested in a shorter period compared to those treated with Xterm. The period from elimination to reinfestation also were significantly longer for premises that were baited with AG bait, compared to those baited with IG bait only. This may suggest that a multitude of factors could affect reinfestation including the presence of neighboring colonies and the sites, rather than just the bait deployment strategies or toxicant alone.

The evolution of termite management in Southeast Asia demonstrated a paradigm shift from conventional soil termiticides to termite baiting strategies. The changes have been driven by regulatory bans on persistent chemicals like chlordane, termite bait introduction, and building owners’ preference for environmentally responsible pest management strategies. Analysis of termite baiting data from 2005 to 2023 highlighted key differences in the baiting period, with Xterm showing the shortest baiting period compared to Sentricon, Exterra, and Exterminex. Despite its effectiveness against *Coptotermes* spp., termite baiting remains inconsistent when applied to fungus-growing termites, necessitating further research into bait formulations tailored against non-heterotermitid species. Reinfestation is a challenge, particularly in public buildings with more complex constructions. The industry’s preference for AG baiting is influenced by 2 major factors, namely (i) the predominantly species, *C. gestroi,* is poorly intercepted in IG monitoring stations, and (ii) Most constructions where people live or work do not or have limited soil area for installation of IG bait stations. Future research should focus on improving bait matrices that could work across diverse termite species and reduce reinfestation risks. Addressing these challenges will be essential to ensuring more sustainable termite management.

## Supplementary material

Supplementary material is available at *Journal of Economic Entomology* online.

toaf081_suppl_Supplementary_Material
