# Supplementary material for: Termite baiting—how it changed the landscape of the pest management industry and termite research in Southeast Asia
Source: J Econ Entomol. 2025 Apr 13;118(3):1029–37. doi: 10.1093/jee/toaf081 (PMC12167846; doi:10.1093/jee/toaf081)
Supplement: toaf081_suppl_Supplementary_Material [file toaf081_suppl_supplementary_material.docx]

**SUPPLEMENTARY FILE**

**Questionnaire survey on termite baiting in Southeast Asia**

**Chow-Yang Lee^1^ and Shao-Hung Dennis Lee**

Department of Entomology, University of California, Riverside, CA 92521, USA

^1^Corresponding author. Email: [chowyang.lee@ucr.edu](mailto:chowyang.lee@ucr.edu)

**Abstract**

A survey of pest management professionals (PMPs) from Malaysia, Singapore, and

Thailand, covering 19,553 baited premises from 2005 to 2023, provides empirical insights into baiting practices, performance, and reinfestation rates. All bait products eliminated termite colonies. The findings reveal significant variations in baiting period (duration to colony elimination), with Xterm outperforming Sentricon, Exterra, and Exterminex. Above-ground (AG) baiting was preferred over in-ground (IG) baiting due to construction constraints and low IG station interception rates. Reinfestation occurred in 9% of baited premises. Bait amount used has slight, but significant impact on the duration to colony elimination.

**Introduction**

Existing literature on termite baiting in Southeast Asia are often based on controlled scientific studies, which may not fully capture real-world practices and the challenges faced by PMPs. To bridge this gap, a questionnaire survey was carried out in June 2023 on termite baiting data provided by pest management companies. It aimed to assess how practitioners implement termite-baiting strategies. This survey offers insights into the practical realities of termite baiting beyond experimental settings.

Twenty-five pest management companies from Malaysia, Singapore, and Thailand that used one of Southeast Asia's major termite bait systems (Sentricon, Xterm, Exterra, and Exterminex) were contacted. Of the 25 companies contacted, 14 companies agreed to participate in the study.

**Materials and methods**

In the survey, each participating pest management company was provided an Excel file to tabulate termite baiting data from 2005–2023. They were asked to review their termite bait account records from 2005 and enter the information into the Excel file. Each data entry is a baited premise. Among the variables surveyed in this study were types of building (landed houses, commercial buildings, public buildings, apartments/ condominiums), baiting method (AG, IG, or combination of both), bait products used (Sentricon, Xterm, Exterra, Exterminex), the amount of bait used to achieve colony elimination, percentage of in-ground stations that were intercepted (if used), baiting period (no. days to elimination), whether reinfestation occurred after elimination, how long after elimination that reinfestation occurs, the year that baiting occurred, and lastly, the termite genera baited. A landed house is a residential property where the owner holds full ownership of the building and its land, such as a linked (terraced) house (= townhouse), detached house (=single family home, bungalow, villa), and semi-detached house (=duplex). An apartment/condominium is a residential unit within a larger building, which can be privately owned or rented where tenants share common areas (e.g., hallways, gyms). Commercial buildings are properties designed and used for business, retail, industrial, or professional purposes, such as offices, shopping malls, hotels, factories, or warehouses. Lastly, public buildings are government-owned or funded structures designed for civic, administrative, or community services, such as schools, libraries, courthouses, or town halls.

Since all termite baits eliminated all infestations in the survey, and homeowners and building owners are always concerned about how quickly the termite infestation could be resolved from their property, we define bait performance as the average time taken to eliminate the infestation. Bait performance was analyzed with the following predictor variables: bait (Sentricon, Exterra, Exterminex, and Xterm), bait placement (IG, AG, and both), building (house, apartment, commercial, and public), bait amount used (in g), and year. For each predictor variable (and their respective levels if categorical), the average baiting period (= average time to eliminate the infestation) was calculated with Kaplan Meier analysis in SPSS ver. 29 (IBM Corp, Armonk, NY), treating infestation elimination as the binary event. Hazard ratios were calculated using the Cox proportional hazards model. For categorical predictors, one level was selected as the baseline: Sentricon (for bait), IG (for bait placement), and house (for building). For each bait, the influence of bait placement and building type on the baiting period was examined separately with Kaplan Meier analysis and Cox proportional hazards models. IG bait and House were used as the baseline levels for comparison. Predictor variable combinations with insufficient sample sizes were excluded from the analysis.

Reinfestation was analyzed with bait (Sentricon, Exterra, Exterminex, and Xterm), bait placement (IG, AG, and both), building (house, apartment, commercial, and public), bait amount, and year as predictor variables. Reinfestation was regarded as a binary event, and the time (days) until reinfestation was used to compare infestation risk associated with predictor variables. Because the reinfestation monitoring times for each premise were entirely dependent on the length of the service contracts, only cases where reinfestation occurred were included in the analyses. The average days until reinfestation were calculated using Kaplan Meier analysis, and hazard ratios were generated using the Cox proportional hazards model with Sentricon, IG, and house as baseline levels for their respective categorical predictors. The influence of bait placement and building type for each bait on days until reinfestation was examined separately with Kaplan Meier analysis and Cox proportional hazards models. Predictor variable combinations with insufficient sample sizes were excluded.

Hazard ratio (HR) can be interpreted as follows: Assuming bait A is used as the baseline level, if HR = 1, it means that termites are being eliminated at the same rate in both bait A and bait B. Hence, there is no difference in period to elimination between the two baits. If HR = 2.0, it means that termites are being eliminated twice as fast with bait B compared to bait A. Lastly if HR = 0.5, this would indicate that termites are being eliminated half as fast with bait B compared to bait A.

**Results**

From the 14 participating pest management companies, data on 19,801 baited premises were received. However, 263 data points were improperly input with missing information – hence, they were discarded in the analysis. Of the 19,553 premises analyzed, 66.7% were from Malaysia, while the remaining were from Thailand (32.7%) and Singapore (0.6%). Almost all baiting (99.1%) was carried out on *Coptotermes*. The baited premises consisted of houses (73.8%), apartments (2.8%), commercial buildings (19.3%) and public buildings (4.1%). Regarding bait usage, 46.8% of the premises were baited with Sentricon, 50.3% were with Xterm, 0.6% were with Exterra, and 2.3% were with Exterminex. Above-ground (AG) baits were used in 80.7% of the premises, in-ground (IG) baits in 15.7%, and the remaining 3.6% were a combination of AG and IG baits. Out of the 3874 IG bait stations used, the interception rate was only 2.1%. The data collected ranged from 2005 to June 2023; however, most data were on premises that were baited between 2018 and 2023 (76.9%). The results for the amount of baits used are as follows: Sentricon (range: 100–1150 g, mode = 100 g, mean ± SEM = 216.7 ± 1.5 g), Xterm (range: 60–2220 g, mode = 120 g, mean ± SEM = 214.7 ± 1.5 g), Exterminex (range: 100–500 g, mode = 200 g, mean ± SEM = 221.7 ± 3.2 g), and Exterra (range: 100–1330 g, mode = 100 g, mean ± SEM = 308.5 ± 25.7 g).

*Baiting period*: Analysis of termite-baited sites revealed significant differences in the baiting period. When averaged across all cases, Xterm had the shortest average baiting period to colony elimination (39.7 days) and a 2.959-fold higher speed of elimination compared to the baseline Sentricon (68.4 days; 95% CI: 67.9–69.0, p < 0.05) (Supplementary Table S1). Exterminex (59.8 days; HR = 1.528, p < 0.05) also had a shorter average baiting period than Sentricon, but was longer than Xterm (Supplementary Table S1). Exterra (65.7 days; HR = 0.982, p = 0.84) was not significantly different from Sentricon (Supplementary Table S1). Amongst bait placement types, AG baiting had the shortest average baiting period (51.6 days) with a 1.175 times speed of elimination when compared to IG placement (HR = 1.175, p < 0.05) (Supplementary Table S1). A combination of both IG and AG baits had a longer baiting period than IG (64.6 days; HR = 0.881, p < 0.05) (Supplementary Table S1). Bait amount (HR = 0.999, p < 0.05) and year of treatment (HR = 1.034, p < 0.05) had a significant impact on the average baiting period (Supplementary Table S1).

When comparing bait placement within bait types, Sentricon AG had the longest average baiting period at 72.5 versus 62.2 days for that of its IG (HR = 0.573, p < 0.05) (Supplementary Table S1). Combined Sentricon AG and IG had an average baiting period of 64.9 days (HR = 0.863 p <0.05) (Supplementary Table S1). Data for Xterm, Exterra, and Exterminex were insufficient to assess the differences between IG, AG, or both placement methods.

Baiting in public buildings required the most extended average baiting period before colony elimination (70.1 days; HR = 0.584, p < 0.05), likely due to structural complexity (Supplementary Table S1). Baiting in commercial buildings (47.5 days; HR = 1.167, p < 0.05) and apartments (40.9 days; HR = 1.563, p = < 0.05) resulted in faster colony elimination versus houses (Supplementary Table S1).

When comparing building types treated with Sentricon baits, treatments in public buildings took 79.7 days (HR = 0.623, p < 0.05), and commercial buildings, took 69.5 days (HR = 0.924, p < 0.05) (Supplementary Table S1). Sentricon treatments in apartments were not statistically different from treatments in houses (63.8 days; HR = 1.160, p = 0.338) (Supplementary Table S1). When Xterm was used, both public (43.8 days; HR = 0.855, p < 0.05) and commercial building (41.6 days; HR = 0.878, p < 0.05), treatments had significantly longer baiting periods compared to the house (35.7 days) (Supplementary Table S1). Treatments for apartments were insignificantly different from houses (38.1 days; HR = 0.975, p 0.665) (Supplementary Table S1). Exterra treatments conducted at commercial buildings had longer baiting periods compared to the house (69.5 vs. 62.0 days) (HR = 0.599, p < 0.05), as did Exterminex treatments (62.7 vs 59.1 days for commercial vs house, respectively) (HR = 0.567, p < 0.05) (Supplementary Table S1). Exterra and Exterminex treatments at apartments and public buildings had too few cases for analysis.

*Reinfestation*: Among 1,794 reinfested cases (9% of total baited sites), Xterm-treated sites exhibited the longest reinfestation-free period (413.2 days; HR = 0.437, p < 0.05), while Sentricon had the shortest (194.9 days) (Supplementary Table S2). Data for Exterra, Exterminex, and Xterm were insufficient and were not analyzed. Use of AG led to longer periods before reinfestation (219.4 days; HR = 0.850, p < 0.05) versus IG (189.7 days), while treatments with both AG and IG placements were insignificantly different from IG (215.0 days; HR = 0.878, p = 0.122) (Supplementary Table S2). Public buildings faced an insignificantly faster speed of reinfestation (166.8 days; HR = 1.250, p = 0.124), as did commercial buildings (198.0 days; HR = 1.047, p = 0.615) compared to houses (207.4 days) (Supplementary Table S2). Reinfestation latency decreased with increasing treatment years (HR = 1.054, p < 0.05) (Supplementary Table S2). The bait amount had no measurable impact (HR = 1.000, p = 0.451) (Supplementary Table S2).

Types of bait had no impact on reinfestation speed for Sentricon-treated sites (Supplementary Table S2). Sentricon treatments at commercial sites had a greater risk of reinfestation compared to house sites (HR = 1.218, p < 0.05), but public buildings were insignificantly different (HR = 1.331, p = 0.052) (Supplementary Table S2).

**Limitation of the survey**

Several weaknesses and limitations were identified in this survey. First, most participating PMPs primarily used either Sentricon or Xterm systems. Hence, there were limited data obtained on Exterra and Exterminex. Second, the time required to eliminate termite colonies was influenced by the PMPs' inspection intervals, typically conducted every two weeks. Additionally, the choice between IG, AG, or combined (both) baiting systems depended on building structure (for example, only AG baits were used when baiting high-rise apartments) and stakeholder preferences. Since home or building owners paid for the baiting services, they were also deciding factor whether IG, AG, or a combination of both was used. Fourth, because the reinfestation monitoring times for each premise were entirely dependent on the length of the service contracts, only cases during the service contract in which reinfestation occurred could be

detected and included in the analyses. Lastly, the bait quantity utilized was calculated based on the total baits deployed without accounting for unused bait remaining in the stations. This suggests potential variability in determining the actual bait consumption during treatment.

**Acknowledgments**

The authors thank the following pest management professionals for their assistance in providing the data in the termite bait survey: Woravuite Wanishsakulpong, Thanakorn Rojrungruang, Juliana Soo, Carol Lam, Regine Lim, Siti Edayu, Richard Ng, John Tan, Andrew Tan, Koay Kean Teik, Hans Lim, Kenny Ler, Jason Jee, Lily Lim, Jerry Hu, Lim Yu Chong, Nor Hisham Badri, Lim Kay Tee.

**Supplementary Table S1**. Kaplan Meier estimates and hazard ratios of termite baiting periods under different treatment variables.

| Predictors | | | n | Average Baiting Period^a^ | 95% CI | Hazard Ratio^b^ | 95% CI | p-value^c^ |
| --- | --- | --- | --- | --- | --- | --- | --- | --- |
| Bait | Sentricon | | 9149 | 68.4 | 67.9–69.0 | - | - | - |
|  | Exterra | | 118 | 65.7 | 58.6–72.7 | 0.982 | 0.819–1.178 | 0.84 |
|  | Exterminex | | 448 | 59.8 | 58.2–61.5 | 1.528 | 1.389–1.681 | < 0.05 |
|  | Xterm | | 9838 | 39.7 | 39.4–40.0 | 2.959 | 2.867–3.054 | < 0.05 |
| Bait Placement | IG | | 3069 | 62.2 | 61.7–62.7 | - | - | - |
|  | AG | | 15771 | 51.6 | 51.2–52.1 | 1.175 | 1.141–1.210 | < 0.05 |
|  | Both | | 713 | 64.6 | 63.6–65.5 | 0.881 | 0.837–0.926 | < 0.05 |
| Building | House | | 14437 | 55.0 | 54.6–55.4 | - | - | - |
|  | Apartment | | 541 | 40.9 | 39.4–42.4 | 1.563 | 1.462–1.670 | < 0.05 |
|  | Commercial | | 3770 | 47.5 | 46.8–48.2 | 1.168 | 1.126–1.211 | < 0.05 |
|  | Public | | 805 | 70.1 | 67.5–72.7 | 0.584 | 0.552–0.618 | < 0.05 |
| Bait x Bait Placement | Sentricon | IG | 3068 | 62.2 | 61.7–62.7 | - | - | - |
|  |  | AG | 5376 | 72.5 | 71.7–73.3 | 0.573 | 0.547–0.600 | < 0.05 |
|  |  | Both | 705 | 64.9 | 63.9–65.8 | 0.863 | 0.795–0.936 | < 0.05 |
|  | Exterra | IG | 0 | - | - | - | - | - |
|  |  | AG | 118 | 65.7 | 58.6–72.8 | - | - | - |
|  |  | Both | 0 | - | - | - | - | - |
|  | Exterminex | IG | 0 | - | - | - | - | - |
|  |  | AG | 447 | 59.8 | 58.1–61.5 | - | - | - |
|  |  | Both | 1 | - | - | - | - | - |
|  | Xterm | IG | 1 | - | - | - | - | - |
|  |  | AG | 9830 | 39.7 | 39.4–40.0 | - | - | - |
|  |  | Both | 7 | - | - | - | - | - |
| Bait x Building | Sentricon | House | 7832 | 67.5 | 67.0–68.1 | - | - | - |
|  |  | Apartment | 42 | 63.8 | 57.5–70.0 | 1.160 | 0.856–1.571 | 0.338 |
|  |  | Commercial | 686 | 69.5 | 67.5–71.4 | 0.924 | 0.853–0.998 | < 0.05 |
|  |  | Public | 589 | 79.7 | 76.6–82.7 | 0.623 | 0.572–0.678 | < 0.05 |
|  | Exterra | House | 74 | 62.0 | 54.6–69.4 | - | - | - |
|  |  | Apartment | 0 | - | - | - | - | - |
|  |  | Commercial | 34 | 69.5 | 67.0–99.5 | 0.599 | 0.394–0.909 | < 0.05 |
|  |  | Public | 0 | - | - | - | - | - |
|  | Exterminex | House | 349 | 59.1 | 57.0–61.2 | - | - | - |
|  |  | Apartment | 0 | - | - | - | - | - |
|  |  | Commercial | 75 | 62.7 | 61.0–64.4 | 0.567 | 0.439–0.731 | < 0.05 |
|  |  | Public | 0 | - | - | - | - | - |
|  | Xterm | House | 6182 | 35.7 | 38.3–39.1 | - | - | - |
|  |  | Apartment | 478 | 38.1 | 36.7–39.5 | 0.976 | 0.889–1.071 | 0.665 |
|  |  | Commercial | 2975 | 41.6 | 41.0–42.3 | 0.878 | 0.841–0.918 | < 0.05 |
|  |  | Public | 203 | 43.8 | 41.1–46.6 | 0.855 | 0.743–0.983 | < 0.05 |
| Bait amount | | | 19553 | - | - | 0.999 | 0.999–0.999 | < 0.05 |
| Year | | | 19553 | - | - | 1.034 | 1.031–1.037 | < 0.05 |
| ^a^ Mean survival time in days. Cases with insufficient sample size are excluded from analyses. | | | | | | | | |
| ^b^ Within each predictor category in reference to the baseline: 'Sentricon' for 'Bait'; 'IG' for 'Bait Placement'; 'House' for 'Building'. | | | | | | | | |
| ^c^ For categorical predictors, hazard ratios are compared to baseline: 'Sentricon' for 'Bait'; 'IG' for 'Bait Placement'; 'House' for 'Building' (α = 0.05). | | | | | | | | |

**Supplementary Table S2.** Kaplan Meier estimates and hazard ratios of time until reinfestation under different treatment variables.

| Predictors | | | n | Average Days Until Reinfestation^a^ | 95% CI | Hazard Ratio^b^ | 95% CI | p-value^c^ |
| --- | --- | --- | --- | --- | --- | --- | --- | --- |
| Bait | Sentricon | | 1706 | 194.9 | 186.5–203.3 | - | - | - |
|  | Exterra | | 2 | - | - | - | - | - |
|  | Exterminex | | 18 | - | - | - | - | - |
|  | Xterm | | 88 | 413.2 | 343.5–482.9 | 0.437 | 0.351–0.545 | < 0.05 |
| Bait Placement | IG | | 804 | 189.7 | 178.4–200.9 | - | - | - |
|  | AG | | 815 | 219.4 | 204.9–233.8 | 0.850 | 0.770–0.938 | < 0.05 |
|  | Both | | 174 | 215.0 | 180.3–249.6 | 0.878 | 0.745–1.035 | 0.122 |
| Building | House | | 1613 | 207.4 | 198.0–216.9 | - | - | - |
|  | Apartment | | 17 | - | - | - | - | - |
|  | Commercial | | 132 | 198.0 | 162.0–234.0 | 1.047 | 0.876–1.250 | 0.615 |
|  | Public | | 49 | 166.8 | 127.2–206.4 | 1.250 | 0.940–1.662 | 0.124 |
| Bait x Bait Placement | Sentricon | IG | 817 | 189.7 | 178.4–200.9 | - | - | - |
|  |  | AG | 736 | 199.4 | 185.7–213.0 | 0.937 | 0.847–1.037 | 0.207 |
|  |  | Both | 174 | 200.6 | 172.0–229.3 | 0.932 | 0.790–1.100 | 0.406 |
| Bait x Building | Sentricon | House | 1547 | 198.4 | 189.5–207.4 | - | - | - |
|  |  | Apartment | 9 | - | - | - | - | - |
|  |  | Commercial | 111 | 164.2 | 133.0–195.4 | 1.218 | 1.004–1.478 | < 0.05 |
|  |  | Public | 48 | 152.5 | 124.0–180.9 | 1.331 | 0.997–1.775 | 0.052 |
| Bait amount | | | 1794 | - | - | 1.000 | 1.000–1.000 | 0.451 |
| Year | | | 1794 | - | - | 1.054 | 1.030–1.078 | < 0.05 |
| ^a^ Mean survival time in days. Cases with insufficient sample size are exluded from analyses. | | | | | | | | |
| ^b^ Within each predictor category in reference to the baseline: 'Sentricon' for 'Bait'; IG' for 'Bait Placement'; 'House' for 'Building'. | | | | | | | | |
| ^c^ Comparison of hazard ratios to the predictor baseline: 'Sentricon' for 'Bait'; 'IG' for 'Bait Placement'; 'House' for 'Building' (α = 0.05). | | | | | | | | |
